# Bycatch mitigation in the West Greenland lumpfish (*Cyclopterus lumpus*) fishery using modified gillnets

**DOI:** 10.1098/rsos.221536

**Published:** 2023-04-05

**Authors:** Søren Post, Flemming Merkel, Zita Bak-Jensen, Christoffer Koch, Rasmus Berg Hedeholm

**Affiliations:** ^1^ Greenland Institute of Natural Resources, 3900 Nuuk, Greenland; ^2^ Department of Ecoscience, Aarhus University, 4000 Roskilde, Denmark; ^3^ DTU Aqua, Technical University of Denmark, 9850 Hirtshals, Denmark; ^4^ DTU Aqua, Technical University of Denmark, 2800 Kgs, Lyngby, Denmark; ^5^ Sustainable Fisheries Greenland, 3900 Nuuk, Greenland

**Keywords:** bycatch reduction, common eider (*Somateria mollissima*), gillnet fishery, seabirds

## Abstract

Bycatch in gillnets is a global issue and mitigation measures that balance target species catch rates, bycatch reduction and fisher support are scarce. In the North Atlantic lumpfish fisheries, bycatch includes marine mammals and seabirds, and there are no permanent technical initiatives to reduce the bycatch. In the West Greenland fishery, common eider bycatch is several thousand individuals annually. We explored if bycatch in this fishery could be reduced by modifying standard lumpfish gillnets by adding a 45 cm high small-meshed net panel to the bottom part of the net. We tested the nets in combination with standard nets and estimated catch rates in a controlled setting in 2021 and in the commercial fishery in 2022. The modified nets had a 71% reduced bycatch rate for common eider and a 25% reduced catch rate for female lumpfish. A combination of the panel and increased seaweed entanglement was the most likely explanation for the effect. In addition to the effect of the net modification, the common eider bycatch decreased significantly during the season, and we recommend studying the net effect further and exploring the option of postponing the fishing season as a simpler way of reducing bycatch.

## 1. Introduction

Incidental bycatch of seabirds is a well-known challenge in gillnet fisheries in all parts of the world, and it is estimated that at least 400 000 birds die annually because of gillnet fisheries [1]. The birds get entangled when diving, making species such as eiders, auks, murres and other seaducks particularly vulnerable. The North Atlantic lumpfish gillnet fisheries are no exception to this problem, with seabird bycatch being reported from all the major fisheries: Norway [2], Iceland [3], Canada and Greenland [4,5]. Besides biological consequences for bird populations, the bycatch has economic ramifications for lumpfish fisheries if the fishing industry fails to maintain MSC certification. Bycatch (including seals) resulted in the withdrawal of the MSC certificate in the world's largest lumpfish fishery in 2019 (Iceland, later re-certified) and a conditioned MSC-certified fishery in the world's second-largest fishery in West Greenland [6]. Hence, minimizing bycatch is in the interest of several stakeholders, including the fishing industry, non-governmental organizations and individual fishers, and should be a conservation objective in all relevant fisheries. Ideally, such measures substantially reduce or eliminate bycatch while at the same time maintaining target species catch rates and adding no unnecessary effort to the fishers. To our knowledge, although several bycatch mitigating studies have shown promising results, e.g. by using light-emitting diodes [7,8], modified buoys [9] or visible net additions [10], no measures have been implemented in any of the larger lumpfish fisheries.

In the North Atlantic lumpfish fisheries, common eider (*Somateria mollissima*) is a key bycatch species [4], and in Greenland, it is by far the most common, with estimated annual bycatch numbers around 10 000–20 000 individuals [5]. Common eider, and other seabirds, forage by the aid of sight along the seafloor at depths down to approximately 40 m [11]. Here, they can encounter lumpfish gillnets set along the seafloor and get entangled. However, it is possible to lower their risk of being caught if the birds’ warning stimulus towards the nets is increased [12]. Birds prone to being caught have colour vision, but their foraging behaviour is mostly linked to low-light conditions (either because of the time of day or water turbidity). Therefore, they should be regarded as seeing only black and white, and the contrast of nets to the surroundings must be as high as possible to reduce the catch [12].

Based on this approach, we explored the possibility of minimizing bycatch (with a focus on seabirds) in lumpfish fisheries and maintaining female lumpfish catch rates by using an easy-to-handle, low-cost, reusable modified version of the standard gillnet. We did this by comparing the catch rates of dominant bycatch species and the targeted lumpfish females between modified and standard nets and evaluated the modified net's applicability in the commercial fishery.

## 2. Methods

### 2.1. Study area and fishery

**Figure 1.**
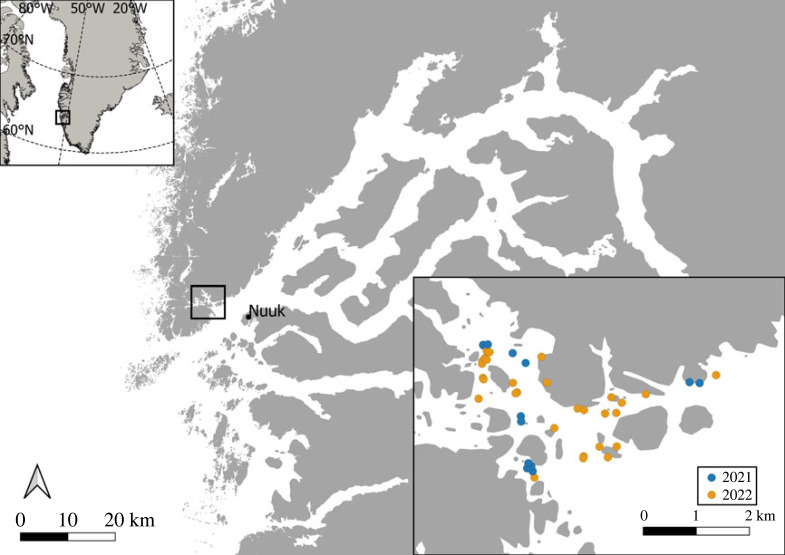
Map of the study area. Lower right insert displays net setting locations by year.

The West Greenland lumpfish fishery extends from approximately 60° N to 70° N (figure 1) and the season typically starts in early April and ends in early June [13]. The fishery uses gillnets set along the bottom in shallow (less than 20 m) nearshore areas from small dinghies typically less than 7 m in length. The nets are typically combined in rows of three–six nets, with the start and end of the net row marked by a small buoy. At each end, a small weight anchors the net row to the bottom. The net soak time is typically 48–72 h, after which nets are emptied, cleaned at sea and reset. During the season, the nets are moved as little as possible. In Greenland, only the roe is used commercially. Hence, carcasses are discarded at sea and males are released alive.

### 2.2. Net and study design

**Figure 2.**
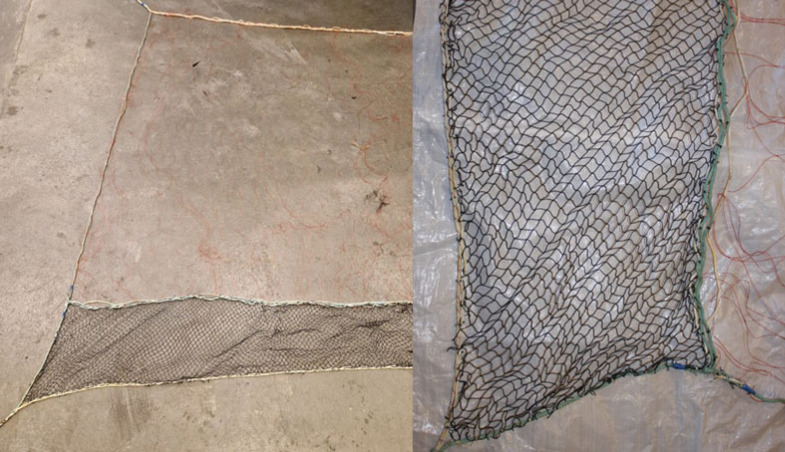
Pictures of the modified gillnet where a small dark meshed net is attached to the bottom part of a standard net. Left: zoomed out. Right: zoomed in on the lower part.

A standard lumpfish gillnet is 60 m long, has a mesh width of 260 mm and is 12.5 meshes high with a twine thickness of 0.52 mm. The net is weighted down by a lead line and a floating top line keeps the net erect. The modified nets were made by adding a small-meshed net to the bottom of a standard gillnet (figure 2). This extra net was 45 cm high and made from black 0.52 mm thick knotless nylon twine with a mesh size of 18 mm.

Two different study designs were used. In 2021, the fishing operations were carried out by the authors, while it was done by commercial fishers in 2022. In both years, the nets were set in the Nuuk area, where the most intense lumpfish fishery in West Greenland takes place (figure 1). To minimize potential differences among local areas, the nets rows were moved one–three times during the seasons. In 2021, the two types of nets were set individually, with a total of 30 modified and 29 standard net settings during the season. In 2022, three–six nets of the same type were combined in rows, as this is common practice in the fishery, with 11 modified rows (41 individual nets) and 20 standard rows (99 individual nets) set during the season.

### 2.3. Sampling

The timing of the fishing season dictated the dates of the experiment. In 2021, the nets were set on 12 April and retrieved on 5 May, while in 2022 set on 13 April and retrieved on 18 May. The nets were attended 10 times in 2021 and eight times in 2022. Date and soak time was noted on each visit, and all specimens were determined to species level and counted. For lumpfish, the sex was also registered. Subsequently, the nets were cleaned and re-set immediately. In 2021, it was noted if common eiders were entangled in the bottom, middle or top of the net, each being one-third of the net height. The fishing procedure followed national laws, ethics and regulations for the Greenland Institute of Natural Resources within Greenland waters.

### 2.4. Analyses

Catch rates were calculated for all species in each net or net row (number caught (24 h)^−1^ (100 m net)^−1^). To compare standard and modified nets, we constructed a generalized additive model (GAM) [14] for the most common species caught. We included species caught in at least 20% of the net settings. This included common eider, male and female lumpfish and Atlantic cod (*Gadus morhua*). The GAM was chosen to allow for nonlinear correlations, which initial data exploration indicated. A Gaussian distribution was used to model the catch of the lumpfish (females and males). Data exploration showed that the catch of common eider and Atlantic cod was zero-inflated and over-dispersed. Therefore, when modelling these two species, we applied a Tweedie distribution, which can cope with these issues [15,16].

In the model, we used ‘net type’ and ‘year’ as factorial variables and ‘soak time’ and ‘yearday’ (day number of year) as continuous variables in individual smooth functions, with restricted degrees of freedom (*k* = 3) [16]. We chose a relatively low k (knots or break points for the smooth functions) because we expected the shapes not to have a wiggly structure. This aligns with a gradually increasing net saturation seen in gillnet fisheries and the dome-shaped signature of the catches in the commercial fishery [13,17,18]. Decreasing catch rates with soak time are seen in multiple gillnet fisheries across species [17–19]. Therefore, we included ‘soak time’ as we expected a reduction in catch rates due to nets getting saturated and becoming more visible with time. We did not model individual saturation curves for the different ‘net type’ as the nets are identical apart from the net addition that catches no fish or birds. Also, including ‘net type’ as a factor implicitly accounts for possible difference caused by the change in net design. We did not include interaction terms in the model. The best-fit model for each species was selected using the Akaike information criterion (AIC) and a backward parameter reduction [20,21]. All analyses and graphical representations were done in R [22] and R-studio [23], using the *mgcv* and *ggplot2* packages [16,24,25]. The full model before model selection can be expressed in R-code as follows:

$$\begin{array}{rl} & \mathrm{gam}(\mathrm{catch}\_\mathrm{pr}\_24\;h\_\mathrm{pr}\_100\;m∼\mathrm{net}\_\mathrm{type}+\mathrm{year}+s(\mathrm{yearday},\;k=3)\\ & \;+s(\mathrm{soak}\_\mathrm{time}\_h,\;k=3),\;\mathrm{data},\;\mathrm{family}=(\mathrm{Gaussian}\;\mathrm{or}\;\mathrm{Tweedie})), \end{array}$$

where ‘catch_pr_24 h_pr_100 m’ are the modelled catch rates and s() is the smooth functions. Finally, for calculating differences in catchability between standard and modified nets, we used the models to predict species-specific catch rates. Statistical significance was inferred at a 0.05 level.

## 3. Results

### 3.1. Female lumpfish

**Figure 3.**
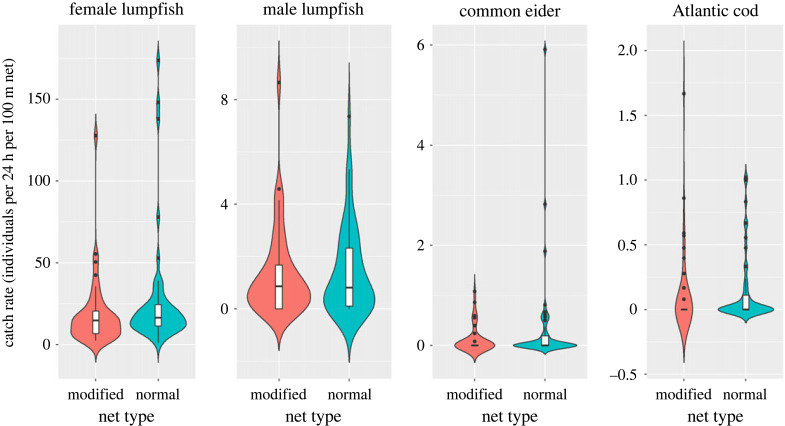
Violin plots comparing observed catch rates for female and male lumpfish, common eider and Atlantic cod in the two different net types.

**Figure 4.**
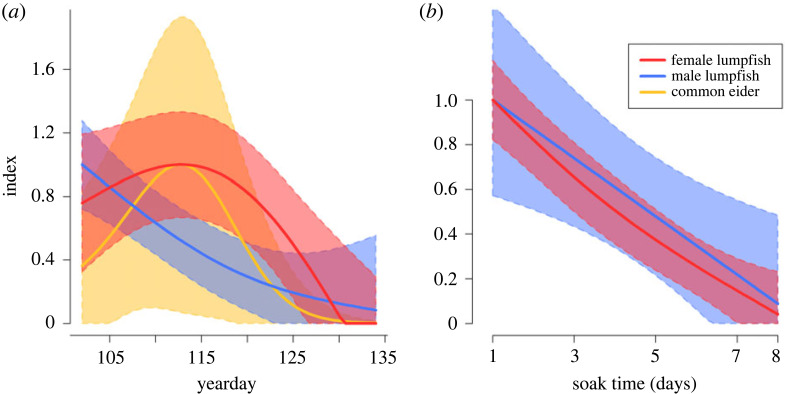
Catch rate index (estimated catch rate relative to maximum) for female and male lumpfish and common eider with 95% CI bands by (*a*) yearday and (*b*) soak time. Parameters from the individual species are only included when significant.

Female lumpfish were caught in all net settings, with 1306 individuals in 2021 and 7253 in 2022, with the large difference being related to a higher effort in 2022 (figure 3) (code and all raw data are provided in the electronic supplementary material, S1–S6). The best-fit model contained all four variables that all had a significant impact on the catch rate (‘soak time’, ‘yearday’ and ‘year’ with *p* < 0.001 and ‘net type’ with *p* = 0.016) (table 1). The modelled mean catch rate was 33% higher in standard nets (38.3 ind. (24 h)^−1^ (100 m)^−1^; 95% CI: 27.1–49.4) than in modified nets (28.9 ind. (24 h)^−1^ (100 m)^−1^; 95% CI 17.7–40.1). No catches were observed in the added small-meshed part of the modified nets. There was an apparent ‘yearday’ effect on the catch rate (figure 4). In the first 20 days of the fishery, the efficiency was high and peaked, but hereafter the catch rate declined rapidly and was reduced to 10% after 30 days. Catch rates declined significantly with increasing ‘soak time’, with an additional day reducing net efficiency by approximately 35% (figure 4).

**Table 1.** Model overview for each species-specific model. Only parameters that were included in the final model for each species are displayed with a *p*-value showing their significance level. Model selection was based on the AIC score.

	species			
parameter	female lumpfish	male lumpfish	common eider	Atlantic cod
net type	*p* = 0.016		*p* = 0.025	
year	*p* < 0.001	*p* = 0.010	*p* = 0.133	*p* < 0.001
soak time	*p* < 0.001	*p* = 0.006		
yearday	*p* < 0.001	*p* < 0.001	*p* = 0.069	

### 3.2. Bycatch

Male lumpfish was the most common bycatch; in total, 504 males were caught (104 in 2021 and 400 in 2022). The best-fit model did not include ‘net type’ (table 1) and the average bycatch rate was 1.4 ind. (24 h)^−1^ (100 m)^−1^ (95% CI 0.9–2). Of note is the distinct change in bycatch rates during the season, where males were very common at the start but disappeared gradually from the catches throughout the season (figure 4).

Common eiders were bycaught in 27 of 90 net settings (19 individuals in 2021 and 58 in 2022). A single individual was caught in 14 of the 27 settings, while two or three individuals were caught in seven of the settings. Four settings caught more than five individuals, all in standard nets. The best-fit model included ‘net type’, ‘year’ and ‘yearday’, but not ‘soak time’ (table 1). The effect of net type on the catch rate of common eider was significant (*p* = 0.025), with presence in both standard and modified nets (37% and 22% of settings, respectively) (figure 3). The mean catch rate in modified nets was 0.15 ind. (24 h)^−1^ (100 m)^−1^ (95% CI 0–0.32) which was a 71% reduction compared with 0.51 ind. (24 h)^−1^ (100 m)^−1^ (95% CI 0.05–0.96) in standard nets. The estimated catch rates peaked at yearday 112 (22 April), with 0.72 ind. (24 h)^−1^ (100 m)^−1^ for the standard nets and 0.21 ind. (24 h)^−1^ (100 m)^−1^ for the modified nets. By the end of the study period, it declined to a minimum of less than 0.001 ind. (24 h)^−1^ (100 m)^−1^ for both nets (still with a 71% difference between the two). The effect of ‘yearday’ was seen as a clear peak in catch rate in the early part of the fishing season simultaneous to the peak in female lumpfish catch rate, but the decline in common eider was steeper than for female lumpfish, and around day 125 (approx. 5 May) the catch rate was reduced to 10% (figure 4). There was no significant difference in where the common eiders were caught in the net (top, middle and bottom) (chi-squared test, $\chi_{2,19}^{2}=3.96$, *p* = 0.14), but overall, the common eiders were least frequently caught in the bottom third of the nets (11%).

The bycatch included a few other common species: Atlantic cod (*N* = 83), Atlantic halibut (*Hippoglossus hippoglossus*, *N* = 18), Greenland cod (*Gadus macrocephalus*, *N* = 15), shorthorn sculpin (*Myoxocephalus scorpius, N* = 7) and spotted wolffish (*Anarhichas minor*, *N* = 6). Rare bycatches included king eider (*Somateria spectabilis*, *N* = 1) and common loon (*Gavia immer*, *N* = 1). On occasion, crabs (*Hyas* spp.) were in some way attached to the net, but these were not systematically counted. One noteworthy difference between net types was that spotted wolffish were completely absent from modified nets.

## 4. Discussion

The modified lumpfish gillnets appeared to have a significant reductive effect on the level of bycatch in the female lumpfish fishery, most noticeably on common eider. The modified gillnets had a 71% lower common eider catch rate and there were no incidents of large (greater than five individuals) catches. Hence, in isolation, the modified nets could potentially reduce common eider bycatch substantially. However, the catch rate of female lumpfish also declined in the modified nets (25%) and to maintain current catches the effort would therefore have to increase. With that effort increase and all else being equal, the bycatch reduction would be approximately 61%, which is still substantial.

The reduction in female lumpfish catch rates naturally reduced the fisher's appreciation of the modified nets. Moreover, the commercial fishers expressed frustrations with the modified nets as they thought the nets caught more seaweed, which is disadvantageous for the fishery and increases handling time and level of frustration. However, we did not investigate the seaweed catch and cannot evaluate the scale of it. In addition to the unbeneficial catch rates in the modified nets, fishers do generally not consider common eider bycatch problematic as the individual fisher catches few and is not concerned with the large-fleet effect. The common eiders are kept for personal consumption and often regarded as a welcome addition to the catch. Therefore, future initiatives to implement the modified nets cannot be expected to have general fisher support. However, the annual common eider bycatch in this fishery is estimated to be as high as 20 000 birds for all of Greenland and it has a pronounced effect on the total Greenland winter population, which is a mixture of birds breeding in Canada and Greenland [5]. Therefore, relatively simple initiatives that potentially reduce the bycatch substantially should be considered from a conservation perspective.

This study did not investigate the underlying reason for the ‘net type’ effect on catch rates. However, common eiders (and many other seabirds) forage along the sea floor with visual navigation [12], so perhaps the more visible panel worked as intended, with the common eiders seeing it at a distance and avoiding it. Additionally, if eiders do not see it, the initial net encounter will be with the small-meshed addition, which will probably deter the birds. When encountering the lower added part, the risk of getting entangled is low (no birds were entangled in the net addition). Ideally, when an encounter happens, the birds will change direction horizontally and away from the net. On the other hand, if the avoidance response is to swim up, they may instead get entangled in the lumpfish net above. The latter, however, seems less likely because the birds were more frequently bycaught in the standard nets. The largest bycatch events, with greater than five individuals, were all in standard nets. We have no clear explanation of why, but eiders flocks may collectively increase the chance of seeing the modified nets, thereby limiting the bycatch to individual birds that fail to notice the nets.

The fishers in our study complained that the modified nets were prone to seaweed entanglement. Therefore, an alternative reason for the lower catch rate could be that seaweed acted as the visual deterrent instead of the net itself. The seaweed build-up (in combination with fish accumulation) may also explain the significant effect of ‘soak time’ on female lumpfish catch rate, again by deterring the fish from approaching the net or the net fishing less effectively. The apparent saturation of the gillnets with soak time aligns with observations in other gillnet fisheries, both for lumpfish [26] and other fish species [18,19]. This result highlights that the current stock assessment, using catch per unit effort from the commercial fishery [13], should ideally account for a soak time effect.

To deter visually oriented foragers (like diving common eider), [12] recommended attaching high-contrast panels to gillnets. Therefore, to improve the net design and address the fisher's concerns, we suggest reducing the height, increasing the mesh size of the added net panel, and adding a visual deterrent in the upper parts of the nets, as this was where most of the common eiders were caught. This deterrent could be a white string woven into the net and should not be an addition that further increases the water pressure in the net, as this may have caused increased seaweed capture.

The seasonal decline in bycatch rate could be used as an alternative and less invasive tool to reduce common eider bycatch. Common eiders migrate towards their summer nesting areas outside the lumpfish fishing area around 1 May (day of year approximately 121 in figure 4) [27], explaining why bycatch rates declined rapidly hereafter in this study. This means that postponing the start of the fishing season by two weeks could have a larger effect on the total bycatch than using the modified nets. The female lumpfish catch rates also declined late in the season, but to a lesser extent. There is little data on the abundance of roe-bearing females in May and June, but late in the season, an increasing number of the caught females are spent either partially or fully (personal observation). Moreover, the commercial value of the roe decreases late in the season, and a postponed season could thus have a large impact on the fishers’ potential income. Therefore, we strongly encourage investigating how a delayed start to the season would affect the catch rates both on bycatch and roe-bearing females and the fleet's possibility of catching the female lumpfish quota. Temporal timing of the fishery may be the optimal approach to reduce the bycatch of common eider in this fishery while maintaining female lumpfish catch rates and having fisher support. We show a significant negative effect of ‘yearday’ on female lumpfish catch per unit effort. This suggests that fishing later in the season is less efficient. However, the declining catch rate could also be a simple effect of local depletion caused by fishery [28]. Tagging studies combined with detailed catch rate information and possibly a local trial with a postponed fishery start could be done to separate effects.

Male lumpfish was the most common bycatch numerically, but males were most often released alive with a minimum handling time out of the water (less than 30 s), and albeit from a small local consumption, the direct fishery-induced mortality is low. Lumpfish are robust, and although the post-capture mortality was not estimated directly, the very active behaviour following release (personal observation) also suggests a very low indirect mortality with minimal population effects.

The most pronounced effect of the gillnet modification was the complete lack of spotted wolffish as bycatch in the modified nets. Wolffish forage strictly along the seafloor, and the small-meshed net addition does not capture these large fish. The overall annual bycatch of spotted wolffish in the lumpfish fishery is estimated at less than 200 tons [5] but while this presumably has little impact on the stock it does illustrate the effect net modification can have, if implemented correctly. Atlantic halibut has been reported as a bycatch in the Greenlandic lumpfish fishery [5]. It is IUCN red listed as ‘Endangered’ globally and should be given particular conservation considerations [29]. In this study, there was no consistent difference in Atlantic halibut bycatches between the net types, as seen for wolffish. Generally, bycatches of Atlantic halibut were too small to allow for any firm conclusions regarding the effect of net modification on catch rates.

The current study focuses on a relatively small local area, and because the spatial and temporal overlap between the lumpfish fishery and common eiders (and other bycatch species) may differ between regions, the modified nets should ideally be tested in other locations. Such investigations should not necessarily be limited to West Greenland waters as bycatch of seabirds is a general issue across North Atlantic lumpfish fisheries, and the common eider is the most common species [4]. Also, bycatch in gillnets is a global issue [1], and effective mitigation measures are few, and all initiatives that offer some level of mitigation should be considered in all gillnet fisheries prone to bycatch.

## Data Availability

Code and all raw data are provided in the electronic supplementary material S1–6 [30].
